# Metalloproteases meprin-ɑ (MEP1A) is a prognostic biomarker and promotes proliferation and invasion of colorectal cancer

**DOI:** 10.1186/s12885-016-2460-5

**Published:** 2016-07-04

**Authors:** Xiao Wang, Jian Chen, Jingtao Wang, Fudong Yu, Senlin Zhao, Yu Zhang, Huamei Tang, Zhihai Peng

**Affiliations:** Department of General Surgery, First People’s Hospital, Shanghai Jiao Tong Univerisity, 85 Wujin Road, Shanghai, 200080 China; Department of Pathology, First People’s Hospital, Shanghai Jiao Tong Univerisity, 85 Wujin Road, Shanghai, 200080 China

**Keywords:** Colorectal cancer, meprin-ɑ, MEP1A, Prognosis, Cell proliferation, Invasion

## Abstract

**Background:**

Meprin displays multiple functions in both health and disease, due in part to its broad proteolytic activity. In this report, we explored the clinical significance and functional relevance of the expression of meprin-ɑ (MEP1A) in colorectal cancer (CRC).

**Methods:**

The mRNA and protein expression levels of MEP1A in tumor specimens obtained from CRC patients was determined by quantitative real-time PCR and Western blot assay and comparatively paired with adjacent mucosa that presented as normal tissue. ShRNA was used to knock-down MEP1A expression in CRC cell-lines and the effects of dampened expression of MEP1A on the proliferation and invasion were determined by colony formation assays, Cell Counting Kit-8 assays and matrigel invasion assays. Moreover, nude mouse xenograft models were designed to investigate the same effect in vivo. In order to determine whether MEP1A expression correlated with CRC clinicopathologic factors and survival, immunohistochemical staining of a tissue microarray containing 88 paired CRC specimens was performed.

**Results:**

In CRC, enhanced expression of MEP1A was seen. Additionally, both in vitro and in vivo, CRC cellular proliferation and invasiveness was inhibited by dampened MEP1A expression. Several parameters were associated with enhanced MEP1A expression including tumor size (*P* = 0.023), staging of CRC by the American Joint Committee on Cancer (AJCC) (*P* = 0.024), and T (*P* = 0.032) and N stages (*P* = 0.001). Moreover, the expression of MEP1A is an independent prognostic factor for overall survival in CRC (HR 3.643; 95 % CI 0.305-5.842; *P* = 0.007).

**Conclusion:**

MEP1A was not only found to be functionally important, but it might also serve as an important and unique indicator of patient prognosis and therapeutic targeting in CRC.

## Background

Colorectal cancer (CRC) is highly prevalent globally with new cases reportedly exceeding 100,000 annually [1]. Although surgical and adjuvant treatment approaches have improved, malignancy-associated long-term survival remains unsatisfactory because of the recurrence and metastasis of the original tumor [2, 3]. A clinically important objective is the identification of functionally important genes in CRC development and progression, and their utility in prognostic prediction.

Meprin is a member of the astacin family and a zinc metalloendopeptidase that was initially discovered as a protease that was highly expressed at kidney brush border membranes [4] and intestinal epithelial cells [5]. Meprin is comprised of two homologous subunit domains that include the meprin-α (MEP1A) domain and the meprin-β (MEP1B) domain that share 42 % identity in their amino acid sequence.

The broad proteolytic activity of meprin in health and disease displays many functions. Thus, meprin is linked to both cancer and inflammatory conditions [6–8]. MEP1A expression is differentially associated with colorectal, breast, osteosarcoma, among other cancer types, and pancreatic cell-lines [9, 10]. Furthermore, MEP1A is aberrantly secreted to the stroma in the setting of CRC [6]. Additionally, human prostate cancer cell models, also expressed MEP1A where it promoted cellular replication and invasiveness [11]. However, in cancer, the precise substrates and pathogenic role of meprins remains unresolved.

In the current report, we aimed to study the expression and clinical significance of MEP1A in CRC. This was achieved by determining MEP1A expression in CRC specimens, and paired comparative analysis to adjacent normal mucosal tissues. Next, by knocking down MEP1A expression with small hairpin RNA (shRNA), we studied functional relevance of MEP1A expression on in vitro and in vivo cellular proliferation and invasion in CRC. Moreover, we studied associations between MEP1A expression, the clinico-pathological features of CRC, and patient survival outcomes by immunohistochemical analysis of a CRC tissue microarray.

## Methods

### Patients and specimens

Collections of tissue specimens from 36 CRC patients (i.e., 24 male and 12 female; median age, 61.3 years, range 25–86 years) that had undergone tumor resection without chemotherapy or radiotherapy before surgery were conducted between October 2014 and April 2015 at the General Department of Surgery at Shanghai Jiao Tong University Affiliated Shanghai First People’s Hospital (China). Two pathologists confirmed the diagnoses and performed tumor staging according to the guidelines of the American Joint Committee on Cancer (AJCC). All CRC and paired adjacent normal mucosal specimens were collected in the General Surgery Department by protocols that were approved by the Institutional Review Boards of Shanghai Jiao Tong University Affiliated Shanghai First People’s Hospital Medical Center. Written and informed consent was provided by all patients for study participation. The freshly obtained cancer tissues and adjacent normal mucosa located 10 cm from the original tumor site were then immediately cryopreserved in liquid nitrogen and archived at −80 °C prior to RNA and protein extraction.

### Cell-lines and reagents

LoVo, Caco2, RKO and HT29 cells are established CRC cell-lines, and were obtained from the Type Culture Collection of the Chinese Academy of Sciences (Shanghai, China). The healthy human colonic mucosal cell-line, NCM460, was obtained from INCELL (San Antonio, TX, USA). All cell-lines were cultured in a fully humidified atmosphere of 5 % CO_2_ in air at 37 °C. Cell-lines were cultured in DMEM/F12 culture media that was supplemented with 10 % FBS (Gibco, USA), 100 U/mL penicillin and 100 mg/L streptomycin.

### Establishing MEP1A-KD cell-lines

Commercially available MEP1A shRNA constructs (Genechem Co. Ltd., Shanghai, China) were used to silence MEP1A. The MEP1A shRNA duplex sequences were as follows:

KD forward: CCGGCCGGGATGATTATGTGAACATCTCGAGATGTTCACATAATCATCCCGGTTTTTG,

KD reverse: AATTCAAAAACCGGGATGATTATGTGAACATCTCGAGATGTTCACATAATCATCCGG.

LoVo and Caco2 cells were transduced with 5 × 10^5^ transducing units/mL of lentiviral particles. Antibiotic selection (1 μg/mL puromycin) was initiated for 7 days at 24 h immediately after transduction. Consequently, LoVo-sh-MEP1A and Caco2-sh-MEP1A were generated. Cells that were transduced with sh-control (LoVo-sh-control and Caco2-sh-control) were used as controls.

### RNA extraction, reverse transcription PCR and quantitative real-time PCR

Total RNA was prepared from cell cultures, fresh primary tumors and normal mucosa of 36 CRC patients by the TRIzol reagent procedure (TaKaRa, Japan) in accordance with the manufacturer’s instructions. Using total RNA at 2 μg, first strand cDNA was synthesized by using the RevertAid™ First Strand cDNA Synthesis Kit, and following the procedures according to the manufacturer’s instructions (Fermentas, USA). To enable reverse transcription PCR, the following PCR conditions were employed: 30 cycles of 94 °C for 1 min, 55 °C for 1 min, 72 °C for 1 min, and a final extension step at 72 °C for 10 min. Next, 10 μl of the PCR-derived amplicon was resolved by 1 % agarose gel electrophoresis and counter-staining by ethidium bromide (EtBr), with resolved bands quantified by Quantity One software (Bio-Rad, USA).

Quantitative real-time PCR (qRTPCR) assays were performed using 4 μl of cDNA (at a dilution of 1:10) and SYBR green (TaKaRa) in a final volume of 20 μl using the ABI 7900 Real-time PCR System (ABI, USA). The amplification protocol used the following conditions: 95 °C for 2 min, and then 95 °C for 10 s, 60 °C for 30 s, and 72 °C for 30 s, with a final extension at 72 °C for 30 s. Relative quantities of the PCR amplicons were determined by normalizing to GAPDH using the Δ cycle threshold (Ct) values. Each assay reaction was performed in triplicate using the following specific primers:

GAPDH, sense 5’-GGAGCGAGATCCCTCCAAAAT-3’ and anti-sense 5’-GGCTGTTGTCATACTTCTCATGG-3’;

MEP1A, sense 5’- AAAGGCCAAGGAAGTGACCT-3’ and anti-sense 5’-ATGTGGGGCAGAGAGATGAC-3’;

PCNA, sense 5’- CTTCCCGCCGTCCTGTAGC -3’ and anti-sense 5’-CTCCTTCTGCACACATTTGAA-3’;

Ki67, sense 5’-TTCGCAAGCGCATAACCCA-3’ and anti-sense 5’-GCCGGCGCATTTTAGTATTTTG-3’.

### Western blot assays

A RIPA lysis buffer (Beyotime Biotechnology, China) was used to isolate total protein from tissue samples or cultured cells and the BCA protein assay kit (Beyotime Biotechnology) determined total protein concentrations. Equal concentrations of isolated protein (i.e., at 30 μg) were resolved through 10 % sodium dodecyl sulfate-polyacrylamide gel electrophoresis (SDS-PAGE) and electro-transferred to a PVDF membrane, which was then blocked for 1 h at room temperature in a 5 % fat-free milk proteins solution that was supplemented with 0.1 % Tween-20. Membranes were reacted with a primary detection antibody (1:500 dilution for MEP1A, R&D Systems, USA and 1:1,000 dilution for GAPDH, Abgent, USA) at 4 °C overnight followed by cross-reaction for 2 h at room temperature with a secondary detection antibody (1:5,000, Abgent) that was directed against goat/rabbit IgG-HRP. To detect cross-reacted proteins, we used an enhanced chemiluminescence (ECL) reagent (Pierce Biotechnology, USA). Quantification by grayscale analysis was performed by employing Quantity One software program analysis (Bio-Rad, USA).

### Plate colony-forming assays and CCK-8 assays

The plate colony-forming assay measures cell proliferation, and follows the recommendations set out in the manufacturer’s instructions. Briefly, 800 log-phase cells were seeded into six-well tissue culture plates then subsequently incubated at 37 °C under a fully humidified atmosphere of 5 % CO_2_ in air for two weeks. Cells were then fixed for 15 min in methanol and stained with Giemsa solution for 20 min. Colonies were then counted and images recorded by standard photography. Independent assays were performed in triplicate. For Cell Counting Kit-8 (CCK-8), cells were seeded in triplicate into 96-well microtiter plates at a density of 2 × 10^3^ cells/well. At appropriate time-points (i.e., 12, 24, 36, 48 h), cells were incubated with CCK-8 reagent (10 μl) for 2 h at 37 °C. Absorbance values were determined at a wavelength of 450 nm using a Gen5 microplate reader (BioTek, USA).

### Scratch assays

Cells were plated in six-well dishes and the scratch assay was conducted. In this assay, LoVo cells were incubated in endothelial cell culture medium that was supplemented with 5 % FBS. When cells had achieved 80 % confluence, a scratch was made across the adherent cultured cells using a sterile plastic micropipette tip that provoked a single homogeneous wound along each well, following which, displaced cells were removed by two PBS washes. Cells were cultured for 36 h and microscopic images were recorded using an ocular grid. Three wounds were samples for each treatment. The relative wound area was calculated as previously described [12].

### Matrigel invasion assays

For this procedure, 24-well Transwell plates with pore diameters of 8 μm (Corning, New York, NY, USA) were pre-coated with a Matrigel matrix (BD Biosciences, San Diego, CA, USA), following which 1 × 10^5^ transfected LoVo cells were suspended in 200 μL of 1 % FBS supplemented RMPI 1640 media, and then seeded to the upper compartment of each inserted well.

Normal growth medium that was supplemented with 10 % FBS, was then added to the lower wells. Cells were allowed to migrate for 48 h at 37 °C, following which, those that had migrated were fixed for 5 min in 10 % methanol and then air dried at room temperature. The invaded cells that were present on the lower surface of the membrane were stained with 2 % crystal violet for 5 min, and then scored by visual inspection and standard light microscopy. To minimize bias, at least five independent fields of view at × 100 magnification were scored following which, the aggregated counts were averaged.

### Nude mouse xenograft models

Xenografts of CRC were established in male BALB/C nude mice (aged 4-weeks) that were provided by the Institute of Zoology, Chinese Academy of Sciences of Shanghai. Briefly, Luciferase tagged sh-MEP1A and sh-control LoVo cells were suspended in 100 μL of PBS at a density of 3 × 10^6^ and adoptively transferred to the above described recipient mice. Half of the mice were subcutaneously adoptively transferred and the remainder were adoptively transferred through the lateral tail vein. Following anesthesia, mice were treated at weekly intervals with D-luciferin (150 mg/kg) by i.p. injection, and imaged 10 min later by an IVIS Illumina System (Caliper Life Sciences). Ten weeks after the tail vein injection, mice were euthanized and examined for the presence of both subcutaneous tumors and lung metastases. All animal procedures strictly followed the Shanghai Jiao Tong University Affiliated Shanghai First People’s Hospital Animal Care and Use Guidelines. All humane efforts were made to minimize animal suffering.

### Immunohistochemistry on tissue microarrays

Since the 36 paired tissue specimens were collected only one year ago and their follow-up data was lacked, we chose to use a tissue microarray (TMA) with an over 5 years’ follow-up data and a detailed clinico-pathological data to determine whether MEP1A expression correlated with CRC clinico-pathological factors and survival. The TMA containing 88 paired CRC specimens was obtained from the Xin Chao Company (Shanghai, China). Tumors were resected between July 2006 and May 2007, with a final follow-up of August 2012. In surviving patients, the median follow-up time was 46.62 months, and a range of 3–73 months. Samples were obtained from 46 male and 42 female patients with a mean age of 68.72 years; range, 24–90 years. Tumor staging was completed by following the AJCC staging criteria [13]. Detailed information on the demographics of the recruited patients was collected (Table 1). Dewaxing of sections by xylene treatment was followed by rehydrating specimens in a graded series of ethanol concentrations followed by antigen retrieval with 0.01 M sodium citrate buffer (pH 6.0).

**Table 1:** Relationship between clinicopathologic parameters and MEP1A or E-cadherin protein expression (*n* = 88)

Parameters	Total	MEP1A		*P*	E-cadherin		*P*
		Low37	High51		Low55	High33	
Age							
<65	31	13	18		16	15	
≥65	57	24	33	0.988^a^	39	18	0.120^a^
Gender							
Male	46	18	28		29	17	
Female	42	19	23	0.562^a^	26	16	0.912^a^
Location							
Right	37	15	22		21	16	
Others	51	22	29	0.808^a^	34	17	0.343^a^
Tumor size (cm)							
<5	47	25	22		31	16	
≥5	41	12	29	0.023*^a^	24	17	0.473^a^
AJCC stage							
I + II	52	27	25		26	26	
III + IV	36	10	26	0.024*^a^	29	7	0.004*^a^
T stage							
T1 + T2	9	7	2		6	3	
T3 + T4	79	30	49	0.032*^b^	49	30	1.000^b^
N stage							
N0	54	30	24		29	25	
N1 + N2	34	7	27	0.001*^a^	26	8	0.032*^a^
M stage							
M0	86	37	49		53	33	
M1	2	0	2	0.507^b^	2	0	0.526^b^
Differentiation							
Well + Moderate	78	35	43		51	27	
Poor	10	2	8	0.182^b^	4	6	0.167^b^
Vascular invasion							
No	84	37	47		51	33	
Yes	4	0	4	0.135^b^	4	0	0.292^b^

Immunohistochemistry on tissue microarrays containing 88 paired CRC specimens was performed and more specific information was given in ‘Methods’

Key: *indicates *P* < 0.05. ^a^ Chi-square test. ^b^ Fisher’s exact test

A MEP1A-specific primary antibody (at a dilution of 1:200; R&D Systems Inc, USA) or an E-cadherin specific primary antibody (1:100; Santa Cruz, USA) was used for immuno-histochemical staining assays. Primary antibody staining was immediately followed by detection with an HRP-conjugated secondary antibody (Dako Cytomation, Glostrup, Denmark). Two specialists blinded to patient outcome provided independent evaluation of the staining. For both MEP1A and E-cadherin, scoring of the staining intensity followed a system of semi-quantitative criteria as described here: 0 (negative), 1 (weak), 2 (moderate), and 3 (strong). The extent of staining was scored as 0 (0 %), 1 (1–25 %), 2 (26–50 %), 3 (51–75 %), and 4 (76–100 %) according to the percent frequency of positively stained cells. Additionally, multiplying the scores of the staining intensity with that of the extent of staining gave “the final staining score.” According to the final scores, two groups were derived; i.e., 1) a low scoring group (0–6), and 2) a group that scored high (7–12).

### Statistical analysis

The Student’s two-tailed *T*-test determined the statistical significance of in vitro and in vivo data. In addition, the two-tailed *χ*2 test and Fisher’s exact test determined the statistical significance of covariate differences. The Kaplan–Meier method determined patient survival rates, and comparisons of survival curves was done using the log-rank test. For experimental variables, the Cox proportional hazards model was used to determine multivariate Hazard Ratios (HRs). An alpha value of *P* <0.05 was considered a statistically significant difference. All statistical analyses were determined by the SPSS version 19.0 statistical software package (SPSS Inc., Chicago, IL, USA).

## Results

### Aberrant MEP1A over-expression in CRC tissues

**Fig. 1:**
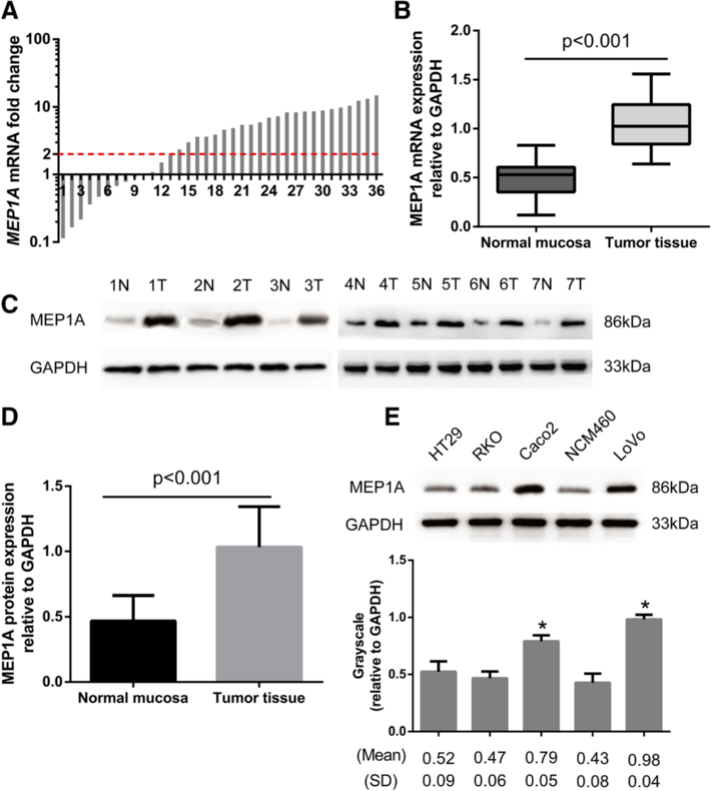
Expression of MEP1A in human colorectal cancer tissues and cell-lines. Relative expression of MEP1A mRNA in 36 paired tumorous samples compared with normal samples. Fold change was calculated by 2^-ΔΔCT^ method (**a**). The mean MEP1A mRNA expression in these 36 tumor tissues was significantly higher than paired adjacent normal mucosa specimens (**b**). Western blot analysis of MEP1A expression in seven paired colorectal tumor tissues (**c**). MEP1A protein is higher expressed in tumor tissues than in paired adjacent normal mucosa (**d**). MEP1A protein expression in five colorectal cell-lines. Grayscale values were evaluated (*n* = 3, * *p* < 0.05, compared with NCM460 cell-line) (**e**)

Of the 36 randomly selected paired cases that were assessed for mRNA and protein expression of MEP1A, 23 (64 %) cases of CRC displayed at least a two-fold increase in MEP1A mRNA levels as compared their adjacent non-malignant tissue counterparts (Fig. 1a). The mean MEP1A mRNA expression levels in tumor tissue specimens was significantly higher than which in paired adjacent normal mucosal specimens (i.e., 1.04 ± 0.04 vs. 0.49 ± 0.03, respectively at *P* < 0.001 by Student’s *t*-test; Fig. 1b). Subsequent Western blotting analysis confirmed that the protein expression levels of MEP1A were also higher in CRC specimens than were found in the matched adjacent non-tumor tissues (i.e., 1.04 ± 0.05 vs. 0.47 ± 0.03 at *P* < 0.001, Student’s *t*-test; Fig. 1c-d). Protein expression of MEP1A in Caco2 and LoVo cells exceeded that found in NCM460 cells (Fig. 1e), which suggested enhanced expression of MEP1A in CRC.

### Knock-down of MEP1A expression inhibits CRC cell proliferation in vitro

**Fig. 2:**
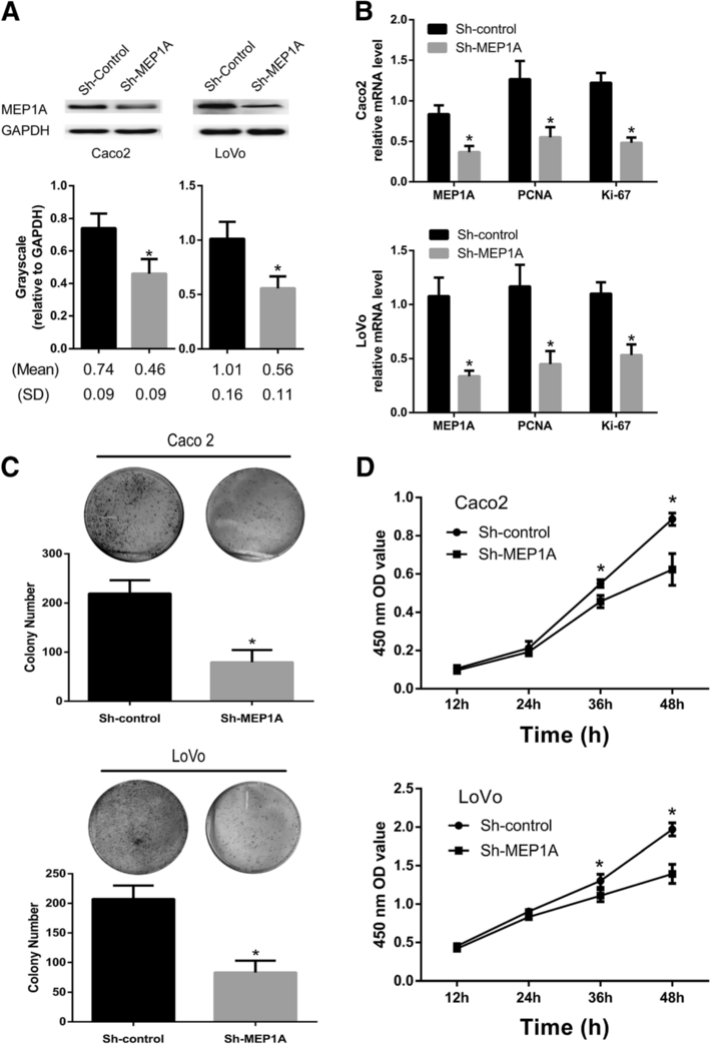
MEP1A knock-down inhibits colorectal cancer cell proliferation. Western blot analysis of MEP1A protein expression in stable knock-down Caco2 and LoVo cell-lines. Grayscale values were evaluated (*n* = 3, * *p* < 0.05) (**a**). Expression of proliferation-related genes was inhibited in MEP1A knock-down cells according to real-time PCR analysis (*n* = 3, * *p* < 0.05) (**b**). Effects of MEP1A knock-down on cell growth was evaluated by plate colony formation assays (**c**) and Cell Counting Kit-8 assays (**d**) (*n* = 3, * *p* < 0.05)

Knock-down of MEP1A expression in transfected Caco2 and LoVo cell-lines was achieved by an MEP1A-shRNA procedure with the goal of exploring how MEP1A expression influences CRC cell proliferation. Western blot assays confirmed the efficiency of MEP1A knock-down (Fig. 2a). In addition, quantitative PCR detected the expression of the proliferation-related genes PCNA and Ki67 to determine the effects of MEP1A knock-down on CRC cell proliferation (Fig. 2b). PCNA and Ki-67 mRNA expression was down-regulated in sh-MEP1A cells (*P* < 0.01). Next, the colony formation and CCK-8 assay were used to determine cell colony forming ability and viability (Fig. 2c, d). Knocking down MEP1A expression in CRC cells consistently dampened colony forming ability as compared sh-control cells (*P* < 0.01). Furthermore, MEP1A knock-down was associated with significantly decreased cellular proliferation as compared with cells that were transfected with control-shRNA (Fig. 2d). These observations suggested a critical role for MEP1A in CRC cellular proliferation.

### Dampened MEP1A expression decreases CRC cell migration and invasion in vitro

**Fig. 3:**
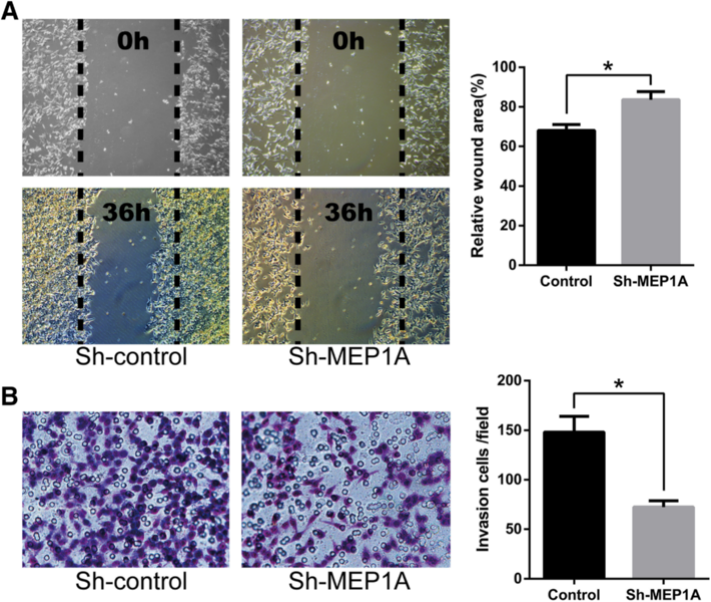
MEP1A knock-down inhibits colorectal cancer cell migration and invasion. Scratch assays and matrigel invasion assays showed that MEP1A knock-down LoVo cells had less migration ability (**a**) and invasion ability (**b**) than control LoVo cells. The wound areas were measured 36 h post injury. The results represent mean ± SD of at least 12 wounds and were analyzed by the Student’s *t*-test (* *p* < 0.05)

**Fig. 4:**
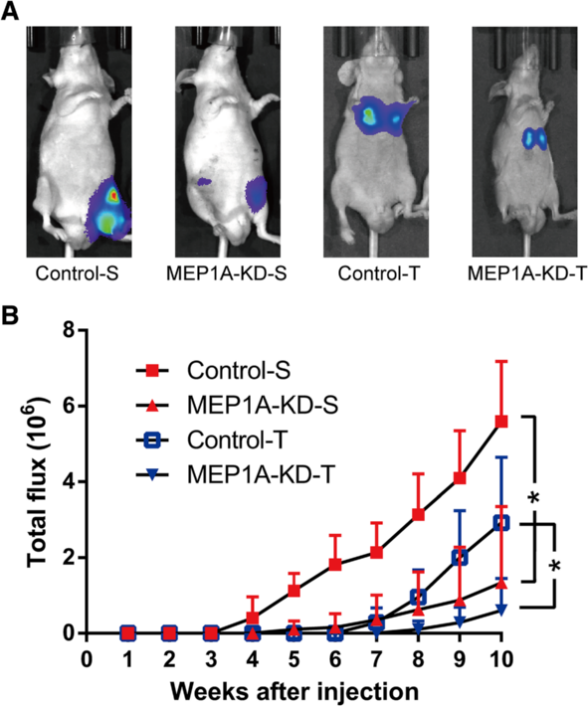
MEP1A knock-down repress colorectal cancer growth and metastasis in vivo*.* Luciferase tagged sh-control and MEP1A-KD LoVo cells (3 × 10^6^) were injected into the 4-week-old nude mice. Half of the mice were subcutaneously injected (Control-S and MEP1A-KD-S, 5 mice each group) and the remainder were injected by lateral tail vein (Control-T and MEP1A-KD-T, 5 mice each group). All four groups’ representative images of luciferase signals (**a**) and photon flux (**b**) are shown (* *p* < 0.05)

To determine the effects of knocking down MEP1A expression on migration and invasion of CRC cells, scratch and matrigel invasion assays were performed using LoVo cells (Fig. 4a and b). The migration of cells into the scratch region was reduced in MEP1A knocked-down LoVo cells (Fig. 3a). Knock-down of the MEP1A protein resulted in a 51.35 % reduction in the invasive ability of LoVo cells (i.e., 72 ± 6 vs. 148 ± 16; cells per field; Fig. 3b). These results indicated that CRC cellular migration and invasion in vitro might be potently augmented by functional MEP1A expression.

### MEP1A knock-down represses CRC growth and metastasis in vivo

Taking the above observation into account, we determined the tumorigenicity of sh-MEP1A and sh-control LoVo cells when adoptively transferred by subcutaneous or lateral tail vein injection into nude mice. All mice survived the procedure and appeared healthy. Formation of tumors was determined by longitudinal in vivo imaging of LoVo cells that stably expressed a luciferase marker gene for a 10 week procedure (Fig. 4a and b). Mice that were adoptively transferred with sh-MEP1A cells exhibited latent tumor morbidity and blunted tumor growth in both the subcutaneous and the tail vein injection groups as compared mice that were adoptively transferred with sh-control cells. These in vivo data were consistent with the in vitro results, and confirmed that MEP1A knock-down repressed CRC growth and metastasis.

### Correlation between MEP1A expression and clinicopathologic factors in CRC

**Fig. 5:**
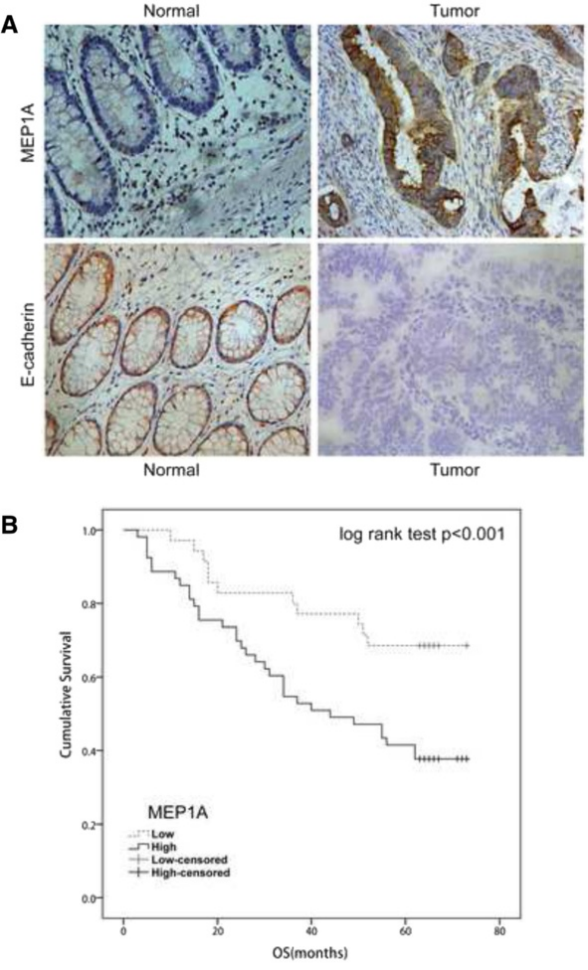
Immunohistochemical staining in colorectal cancer and Kaplan-Meier plots with log rank test of overall survival (OS). Adjacent normal mucosa showing very weak MEP1A staining (upper left, 200×), and high staining of MEP1A (upper right, 200×) in a moderately differentiated colon tumor. On the contrary, E-cadherin showing high expression in normal mucosa (lower left, 200×) while very weak staining in poorly differentiated tumor tissue (lower right, 200×) (**a**). Overall survival of 88 patients in relation to MEP1A expression levels as determined by immunohistochemical staining of tissue microarrays. OS was significantly lower in patients with tumors that expressed high levels of MEP1A as compared patients with tumors that expressed low levels of MEP1A (**b**)

MEP1A and E-cadherin expression was determined by immunohistochemical analysis of a TMA containing 88 CRC specimens and paired adjacent normal mucosa. Membrane-restricted expression of MEP1A was seen with negligible extracellular staining, while E-cadherin expression was restricted to the membrane (Fig. 5a). Tumors showed variable (i.e., weak, moderate, and strong) MEP1A expression. A high level of MEP1A expression was detected in 51 of 88 CRC specimens. Associations of MEP1A and E-cadherin expression with clinicopathological factors are shown (Table 1). High expression of MEP1A was significantly correlated with tumor size (*P* = 0.023), AJCC stage (*P* = 0.024), T stage (*P* = 0.032) and N stage (*P* = 0.001). Low E-cadherin expression (i.e., in 55 of 88 specimens) was markedly associated with AJCC stage (*P* = 0.004) and N stage (*P* = 0.032). In most cases, tumors with high MEP1A expression showed low E-cadherin expression. A negative correlation shown between expression of MEP1A and E-cadherin was identified by Spearman’s rank correlation coefficient (*P* < 0.001; Table 2).

**Table 2:** The association between MEP1A and E-cadherin expression

Tissue sample	E-cadherin expression		*P* value	r
	Low	High		
MEP1A Low	14	23		
MEP1A High	41	10	<0.001	−0.43

### MEP1A or E-cadherin expression – Analysis of survival and prognosis

Kaplan-Meier analysis with a log rank test for overall survival (OS) determined possible associations between tumor expression of MEP1A and patient survival (Fig. 5b). Patients that displayed high levels of MEP1A expression in their tumor had a poorer OS (*P* < 0.001) than did patients with low levels of MEP1A expression. Using a univariate analysis in the Cox proportional hazards model, a decreased OS was associated with the following characteristics: tumor location, AJCC stage, LNM stage, distant metastasis, vascular invasion and the expression of MEP1A and E-cadherin (Table 3). The expression of MEP1A was an independent prognostic factor for OS as determined by multivariate analysis (i.e., HR 3.643; 95 % CI 0.305-5.842; *P* = 0.007).

**Table 3:** Univariate and multivariate analysis of overall survival

	Univariate analysis		Multivariate analysis	
	HR (95 % CI)	*P* value	HR (95 % CI)	*P* value
Age				
<65	1			
≥65	1.350 (0.706-2.581)	0.364		
Gender				
Male	1			
Female	0.910 (0.503-1.648)	0.756		
Location				
Right	1		1	
Others	0.470 (0.259-0.852)	0.013*	0.666 (0.335-1.323)	0.246
Tumor size (cm)				
<5	1			
≥5	1.801 (0.993-3.267)	0.053		
AJCC stage				
I + II	1		1	
III + IV	3.106 (1.701-5.671)	<0.001*	1.335 (0.305-5.842)	0.701
T stage				
T1 + T2	1			
T3 + T4	1.662 (0.514-5.370)	0.396		
N stage				
N0	1		1	
N1 + N2	3.106 (1.701-5.671)	<0.001*	2.129 (0.468-9.686)	0.328
M stage				
M0	1		1	
M1	10.801 (2.448-47.658)	0.002*	14.382 (1.059-195.249)	0.045*
Differentiation				
Well + Moderate	1			
Poor	2.026 (0.627-6.548)	0.238		
Vascular invasion				
No	1		1	
Yes	6.188 (2.093-18.295)	<0.001*	1.190 (0.264-5.366)	0.821
MEP1A				
Low	1		1	
High	5.977 (2.651-13.478)	<0.001*	3.643 (0.305-5.842)	0.007*
E-cadherin				
Low	1		1	
High	0.425 (0.215-0.842)	0.014*	0.605 (0.252-1.453)	0.261

Key: *indicates *P* < 0.05

## Discussion

The involvement of the metalloprotease MEP1A in multiple cancers was previously shown [14]. However, studies on MEP1A expression during tumorigenesis and progression of CRC are rare and the molecular mechanisms that implicate involvement of MEP1A in CRC remain unclear.

For the first time, the current study showed that mRNA and protein expression of MEP1A were increased in primary CRC tissues. Immunohistochemistry showed that high MEP1A expression in CRC tissues was significantly aligned to other features of the malignant tumor. These characteristics included tumor size, AJCC stage, tumor depth, and lymph node metastasis, which suggested that expression of MEP1A might serve as a CRC biomarker.

E-cadherin is a transmembrane glycoprotein and the major structural component of adherent junctions of the epithelium that mediate homotypic interactions between adjacent cells that are calcium-dependent. In cancer, decreased E-cadherin expression is functionally important in the progression of well-differentiated adenoma to invasive carcinoma [15], Moreover, E-cadherin might thus serve as a tumor suppressor [16].

In this immunohistochemical study, we found that high MEP1A expression correlated negatively with E-cadherin expression, suggesting that MEP1A is involved in tumor migration and invasion. To confirm this hypothesis, scratch and matrigel invasion assays revealed decreased migration and invasion of LoVo cells in the sh-MEP1A group as compared their control group counterparts.

Studying the effects of MEP1A on CRC cell proliferation in CCK-8 assays revealed that MEP1A knock-down in Caco2 and LoVo cells inhibited their proliferation. Moreover, we noted significantly reduced colony formation in MEP1A knock-down cells. These data collectively suggested that suppressed MEP1A expression blocked cell proliferation and further implied specific therapeutic targeting of MEP1A in the setting of CRC.

Based on these in vitro findings, we proceeded to investigate the tumorigenicity of sh-MEP1A and sh-control cells. As anticipated, down-regulation of MEP1A suppressed both subcutaneous and metastatic tumor formation in nude mice. These in vivo data were consistent with observation made in vitro, and confirmed that MEP1A knock-down inhibited CRC growth and invasion.

The potential utility of MEP1A expression as a prognostic marker in CRC has not been previously reported. In this report, Kaplan-Meier survival analysis demonstrated that high MEP1A expression was significantly related to poor prognosis that is commonly seen in CRC patients following surgical resection (*P* < 0.001). In our study, patients with elevated MEP1A expression were significantly linked to a poorer OS outcome. Furthermore, MEP1A expression was suggested as an independent prognostic factor by Cox regression analysis. These observations suggested that MEP1A might uniquely offer prognostic prediction in the setting of CRC.

It should be noted that there are some limitations of our study. First, there might be a potential selection bias for only patients who had undergone tumor resection without chemotherapy or radiotherapy were included in the mRNA and protein expression study. Second, MEP1A overexpressing cell lines were not investigated in our study in the present day. Third, further studies are required to confirm MEP1A/E-cadherin association and our hypothesis that MEP1A is a potential therapeutic target for CRC.

## Conclusions

We conclude that MEP1A played a functionally crucial role in CRC carcinogenesis, which was particularly evident in terms of tumor proliferation and invasion. For the first time, we show that MEP1A expression was elevated in CRC tissues at both the mRNA and protein levels. Furthermore, cell proliferation and invasion was blocked when expression of MEP1A was knocked down in CRC cells. Moreover, we confirmed a role for MEP1A in CRC progression in relevant animal models. Finally, we provided clinical evidence that suggested MEP1A might serve as an independent prognostic indicator in the setting of CRC outcomes.

## Abbreviations

AJCC, American Joint Committee on Cancer; CCK-8, Cell Counting Kit-8; CRC, colorectal cancer; Ct, cycle threshold; ECL, enhanced chemiluminescence; EtBr, ethidium bromide; MEP1A, meprin-ɑ; MEP1B, meprin-β; qRTPCR, quantitative real-time PCR; SDS-PAGE, sodium dodecyl sulfate-polyacrylamide gel electrophoresis; TMA, tissue microarray
