# Correction to: Metalloproteases meprin-ɑ (MEP1A) is a prognostic biomarker and promotes proliferation and invasion of colorectal cancer

**DOI:** 10.1186/s12885-017-3767-6

**Published:** 2018-01-11

**Authors:** Xiao Wang, Jian Chen, Jingtao Wang, Fudong Yu, Senlin Zhao, Yu Zhang, Huamei Tang, Zhihai Peng

**Affiliations:** 1 Department of General Surgery, First People’s Hospital, Shanghai Jiao Tong Univerisity, 85 Wujin Road, Shanghai, 200080 China; 2 Department of Pathology, First People’s Hospital, Shanghai Jiao Tong Univerisity, 85 Wujin Road, Shanghai, 200080 China

**Fig. 3:**
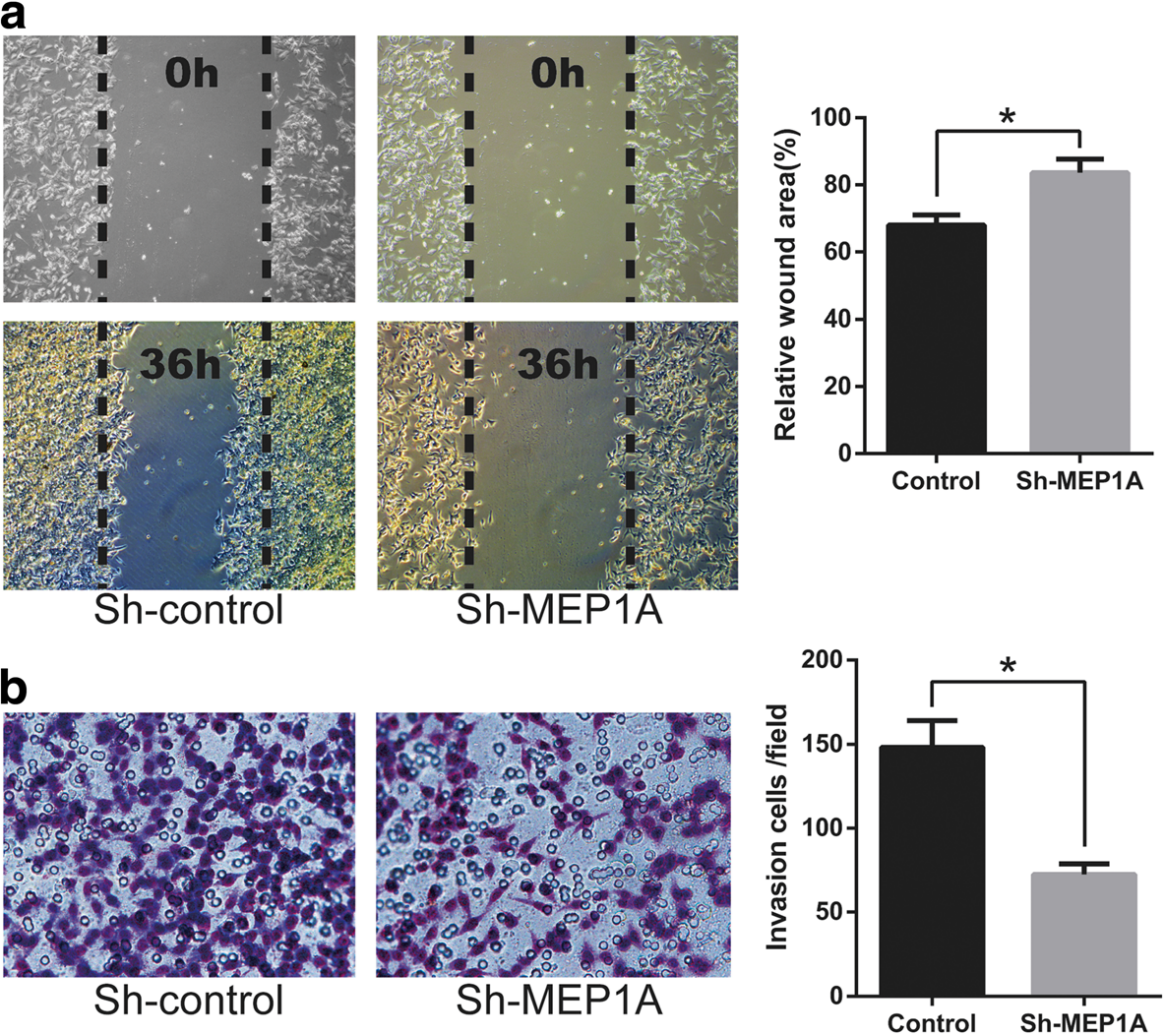
MEP1A knock-down inhibits colorectal cancer cell migration and invasion. Scratch assays and matrigel invasion assays showed that MEP1A knock-down LoVo cells had less migration ability (**a**) and invasion ability (**b**) than control LoVo cells. The wound areas were measured 36 h post injury. The results represent mean ± SD of at least 12 wounds and were analyzed by the Student’s t-test (* *p* < 0.05)
